# Good functional outcome evaluation of free vascularized fibular head graft (FVFHG) as treatment after resection of giant cell tumor (GCT) campanacci 3 at proximal humerus: A case report

**DOI:** 10.1016/j.ijscr.2019.07.075

**Published:** 2019-08-01

**Authors:** Ruksal Saleh, Henry Yurianto, Padlan Pasallo, Astrawinata Guatama, Erich S. Subagio

**Affiliations:** Orthopaedy and Traumatology Department, Hasanuddin University School of Medicine, Makassar, Indonesia

**Keywords:** Giant cell tumor, Free vascularized fibular head graft, Reconstruction, Proximal humerus, Case report

## Abstract

**Introduction:**

Giant cell tumor (GCT) is benign aggressive tumors with a high rate of recurrence and capacity to metastasize. Wide resection is the treatment of choice, but this creates a flaw at the proximal end of the humerus. There are various methods exist as the treatment option to fixed this problem.

**Presentation of case:**

We here present our experience on wide resection and free vascularized autogenous fibula head grafting for GCT at the proximal humerus of a 32 years old male. We performed free vascularized fibular head graft (FVFHG) as a reconstruction method followed by sling procedure and used the long head of biceps tendon. Evaluation of anatomical, functional, and radiological outcomes of this management was performed. After 3 years, the patient has a good outcome.

**Discussion:**

Free vascularized fibula graft is the most favored as a treatment after resection of a tumor on the proximal humerus. The advantages are can be harvested without many difficulties and rapid healing. In our case, we used a free vascularized fibular head graft (FVFHG) for proximal humerus reconstruction after resection giant cell tumor (GCT) on the right proximal humerus. We use the peroneal artery as vascular pedicle due to well vascularity to the peripheral part of fibula. There is no fibula head reabsorption after three years post-operation.

**Conclusion:**

FVFHG for reconstruction modality as the treatment after resection of GCT grade Campanacci 3 on proximal humerus shows satisfactory result following long term evaluation.

## Highlights

• Vascularized fibula could be used to reconstruct wide resected proximal humerus.
• Peroneal artery pedicle ensure vascularization of harvested long graft.
• Rehabilitation for either recipient or donor site is crucial.

## 1 Introduction

Giant cell tumor (GCT) is a rare primary bone tumor that typically occurs in the meta-epiphyseal region of a long bone [1]. It is benign locally aggressive tumor with a high rate of recurrence and capacity to metastasize [[1], [2], [3]]. Wide excision is the management of choice to solve this problem, but this creates a defect [4]. The preferred modalities for the defect reconstruction include vascularized/non-vascularized bone graft, osteoarticular allografts, and custom-made prosthesis. Satisfying target to achieve by orthopedic surgeon team towards limb salvage in upper extremity musculoskeletal tumor must include limb function preservation while maintaining tumor resection and halt recurrence [5]. Limb-sparing procedures has been slowly replacing amputation regarding the technology of radiology diagnostic, microsurgery advancement and more effective adjuvant therapy following surgery [5].

In reconstruction as the treatment after resection of bone tumor at proximal humerus, we prefer to use free vascularized fibular head graft (FVFHG) transfer method. This became the first choice for reconstruction of such defects at many institutions as well as ours due to its safety as well as predictable outcome and able to confidently applied especially in defect of proximal humerus or distal radius [6].

We present our experience on wide resection and modified free vascularized autogenous fibula head grafting for GCT at the proximal humerus of a 32 years-old-male with fair long term outcome evaluation. This case is arranged and reported in line with the Surgical Case Report Guidelines (SCARE) criteria [7].

## 2 Presentation of case

**Fig. 1:**
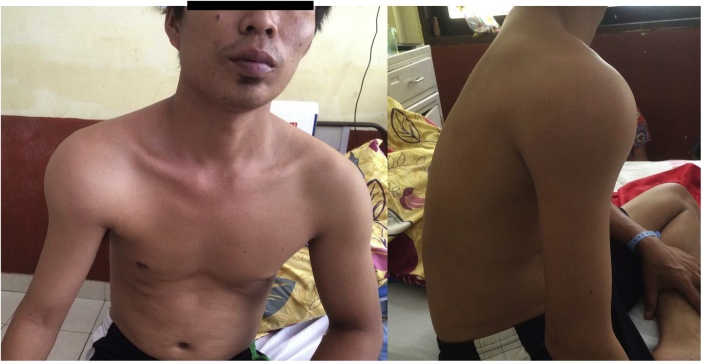
Preoperative clinical finding.

A 32 years-old-male came to the hospital with the chief complaint of a lump at the proximal part of the right arm since 3 months before admitted to the hospital. The lump was as the size of a marble at the beginning and has enlarged as it is now. Pain is felt continuously, irritating and does not radiate. There was no history of trauma, fever and no remarkable past history. No relevant genetic information or family history before. The patient is right-hand dominant and work as a mechanic. From physical examination, we found a lump at proximal humerus, firm, well-demarcated, no discoloration, no wound or ulcer. Patient has a good distal neurovascular (Fig. 1).

**Fig. 2:**
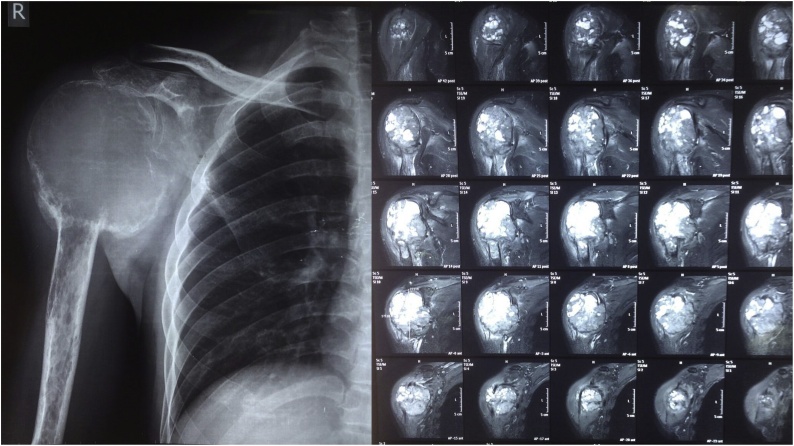
a).Plain radiograph, b).MRI.

His plain radiograph showed an eccentric lytic lesion at epi-metaphyseal proximal humerus which extends to soft tissue, destructs the subchondral and cortical bone of proximal humerus (Fig. 2). Magnetic Resonance Imaging (MRI) showed edema around the lesion with diffuse non homogenous enhancement extended to glenohumeral joint, but no histology examination before surgery was performed. From the radiograph and MRI, we concluded the diagnose was GCT Campanacci 3.

**Fig. 3:**
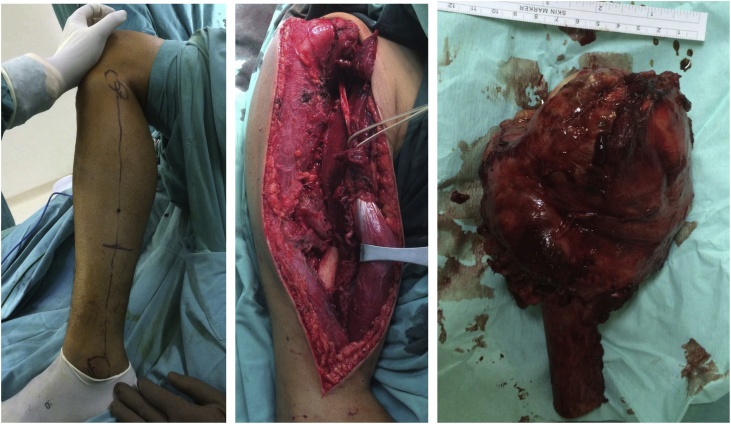
Intraoperative finding.

**Fig. 4:**
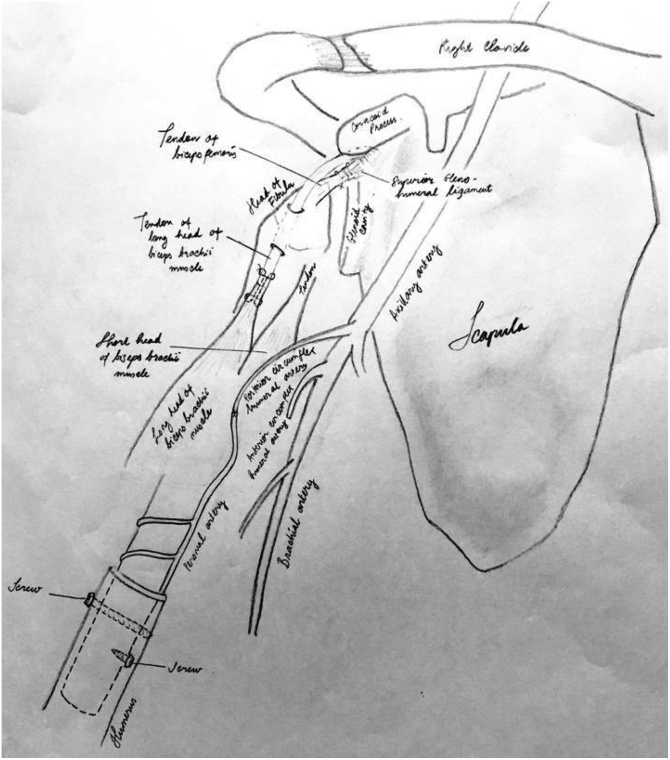
Diagram of operative procedure.

We performed FVFHG as a reconstruction modality (Fig. 3), in which long head of biceps brachii was attached to the fibular head, sutured the bicep femoris tendon to the Superior Glenohumeral Ligament (SGHL) to maintain the continuity and stability of fibular head for the purpose of preserving shoulder joint stability as well [3]. Peroneal vascular (artery and vein) were attached to the circumflex humeral artery and vein to ensure graft vascular supply. Resection of the tumor was followed by FVFHG to the proximal humerus in order to preserve good shoulder stability (Fig. 4). We are not the pioneer for this method but provide a solid successful evidence for this approach. Three years target follow up for anatomical, functional and radiological outcomes evaluation was undergone.

**Fig. 5:**
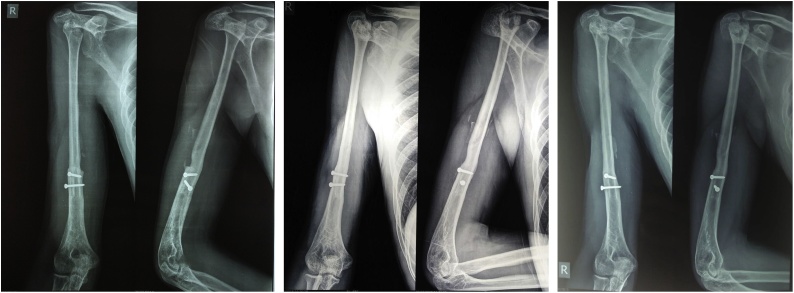
Postoperative plain radiograph: a). 8 months postoperative, b). 2 year post operative, and c).3 years postoperative.

**Fig. 6:**
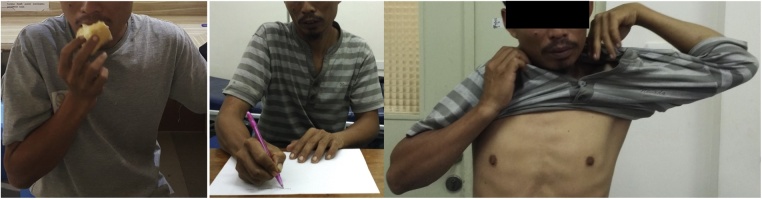
follow up functional outcome after 3 years postoperative.

After 3 years follow up, the patient showed good functional outcome with radiographic union of the graft and no recurrence of tumor (Fig. 5). The patient is currently able to perform daily activities, such as maintain personal hygiene, write, self-feed, hold a glass, grooming, and drive his motorcycle (Fig. 6). The functional outcome rating after reconstruction of the proximal humerus was 80 percent, while score 1 is the worst and 5 is the best for each item using the Musculoskeletal Tumor Society scores [8]. Assessment of pain, emotional acceptance, and manual dexterity from this patient was in scores 5, means satisfactory. We concluded that the functional outcome is satisfactory with no further symptom nor complaint of dexterity.

## 3 Discussion

Upper limb function after resection of GCT at the proximal humerus which depends solely on reconstruction technique are to ensure revascularization, conserve shoulder joint stability and provide good elbow joint function. One of the technique choices was vascularized autograft. The advantages of the vascularized autograft are more rapid healing (due to immediate vascularity), graft hypertrophy and strength to mechanical failure compared to avascularized graft [5]. The fibula is currently the most favored donor for free vascularized transfer since it could be used for segmental defect reconstruction up to 26 cm thanks to its long and straight structure. It can be harvested without many difficulties, and the nutrient artery arises from the peroneal artery [5]. Harvesting procedure itself require a long operation time performed by a skillful microsurgery orthopedic surgeon, not to mention autologous tissue sacrifice.

In our case presentation, the FVFHG for reconstruction modality as the treatment after resection of giant cell tumor on proximal humerus showed satisfactory result following long term evaluation on anatomical, functional, and radiological parameters without any sign of tumor recurrence on three years after surgery. It shows the same with a study performed by Rose et al. [9] that reported the success in limb salvage of the humerus by using vascularized fibula graft. The outcome was excellent, with no loss of limb or local recurrence, and donor site morbidity was low and no complications were found.

Theoretically, the anterior tibial artery is a major blood supplier for proximal epiphysis and the proximal two-thirds of the diaphysis of the fibula. However, in this patient, we chose the peroneal artery as a vascular pedicle because of a long defect up to two-thirds of the humerus or more than 10 cm. It is the same procedure as done by Onoda et al. [6] to their three of eight patients study due to bone defect after tumor resection more than 10 cm. This is because the vascularity of the head and peripheral parts of the fibula is safer by using the peroneal artery as a vascular pedicle in adult patients with defect more than 10 cm [6].

The other advantages by using peroneal artery as vascular pedicle are bone union due to well vascularity to the peripheral part of the fibula was obtained [6]. In this patient, it can only be followed up in the eighth months, when the bone union was occurred. Hypertrophy was seen in three years follow up and no fibular head reabsorption. This is different from the other with the FVFHG study that reported no hypertrophy occurred in all of the study patients [10]. In addition, there are also reabsorption of the fibular head in all patients. ^10^

Donor site morbidity from free vascularized fibula graft including mild pain at the donor site, and some complaints of numbness on the side of the leg and dorsum of the foot. The impaired flexion or extension of the great toe is not uncommon [5]. Feuvrier et al. [11] revealed that they took more cautious approaches during the walk to reduce the risk of falling. An early rehabilitation program is important to improve the physical abilities following the vascularized free fibula harvest. In these patients, no donor site morbidity was found as mentioned above. The patient is recently able to take care of personal hygiene, write, self-feed, hold glass, groom, even drive on his motorcycle. The outcome is satisfactory with no further symptom nor complaint of dexterity.

Some studies observed that there were some predictive values for tumor recurrence from giant cell tumors, including the classification of Campanacci from tumors, surgical methods, the involvement of the cortical bone and the involvement of soft tissue. The recurrence rate in the Campanacci -as an independent recurrence factor- grade 1 group was 0, whereas that in grades 2 and 3 was 13.51% and 41.67%, respectively [12]. Fortunately, there was no recurrence in this patient after a three-year follow-up even though this patient had grade Campanacci 3.

## 4 Conclusion

FVFHG for reconstruction modality as the treatment after resection of GCT grade Campanacci 3 at proximal humerus shows satisfactory result following long term evaluation on anatomical, functional, and radiological parameters without any sign tumor recurrence.

## Declaration of Competing Interest

The authors declare that they have no competing interests.

## Sources of funding

This study was funded independently.

## Ethical approval

This study was approved by the ethical board of Hasanuddin University of Makassar. Our patient has signed terms of consent to participate in the research of this case report. The institutional ethical committee has approved the publication of this case report

## Consent

Written informed consent was obtained from the patient for publication of this case report and accompanying images.

## Author contribution

Ruksal Saleh: concepts, design, surgeon, definition of intellectual content, literature research, clinical studies, data collections, data analysis, manuscript editing & review.

Henry Yurianto: concepts, design, surgeon, definition of intellectual content, literature research, clinical studies, data collections, data analysis, manuscript editing & review.

Padlan Pasallo: concepts, design, surgeon, definition of intellectual content, literature research, clinical studies, data collections, data analysis, manuscript editing & review.

Astrawinata Guatama: concepts, design, definition of intellectual content, literature research, clinical studies, data collections, data analysis, manuscript writing.

Erich Svante Subagio: literature research, clinical studies, experimental studies, data collections, data analysis, manuscript writing.

## Registration of research studies

We are not pioneer of this method as described in page 3 of manuscript

## Guarantor

Padlan Pasallo

Astrawinata Guatama

## Provenance and peer review

Not commissioned, externally peer-reviewed
